# Isobaric mass tagging and triple quadrupole mass spectrometry to determine lipid biomarker candidates for Alzheimer's disease

**DOI:** 10.1371/journal.pone.0226073

**Published:** 2019-12-10

**Authors:** Suzumi M. Tokuoka, Yoshihiro Kita, Takao Shimizu, Yoshiya Oda

**Affiliations:** The University of Tokyo, Graduate School of Medicine, Lipidomics Laboratory, Hongo, Bunkyo-Ku, Tokyo; Niigata University, JAPAN

## Abstract

The isobaric tagging method widely used in proteomic and lipidomic fields, with the multiple reaction monitoring (MRM) approach using a triple quadrupole mass spectrometer, was applied to identify biomarker candidates from plasma samples for Alzheimer’s disease (AD). We focused on the following phospholipids that have amino groups as the functional group: phosphatidylethanolamine (PE), Lyso-PE, phosphatidylserine, and Lyso-phosphatidylserine. We also investigated fatty acids that have a carboxy group. A sixplex tandem mass tag (TMT) was used for the isobaric tagging method in this study. The TMT reaction had high reproducibility in human plasma. A total of 196 human plasma samples from three AD cohorts were used for the study, and compared to pooled plasma quality control (QC) samples. The described method required only 40 MRM measurements, including the pooled QC samples, for a full comparison of the data. We found that the content of free fatty acids increased in AD samples in all the three cohorts, alkenyl PEs (ePEs) decreased over a one-year interval in AD patients, and ePEs weakly correlated with amyloid peptide (a-beta) 1–42 in cerebrospinal fluid. In conclusion, total free fatty acids in plasma are a risk factor for AD, and ePEs monitor candidates for AD. Therefore, TMT-lipidomics is a powerful approach for the determination of plasma biomarkers because of the high sample throughput.

## Introduction

Plasma samples are widely used in clinical diagnosis because these samples are relatively easy and less invasive to collect from patients and healthy subjects. More importantly, blood collection is possible for almost all patients. Human plasma usually contains a large amount of lipids, whose contents and species vary depending on the health status. Therefore, lipid profiling of serum or plasma samples using mass spectrometry (MS) has been the method of choice for the discovery of diagnostic biomarkers and elucidation of disease mechanisms in many studies. In quantitative analysis, using liquid chromatography (LC)/MS, the stability of ionization is not sufficient, and single sample analysis from multiple injections is carried out. The quantitative variation of each sample is then normalized to the aliquot sample, also referred to as the QC sample[1],[2]. It is a common approach to correct data variation based on repeated analysis of QC samples. However, the biggest problem with this approach is the throughput of sample analysis. It is not realistic to arrange multiple expensive devices in one laboratory; therefore, the measurement throughput is limited by the time required for LC/MS.

In order to increase the throughput in proteomics, Pappin et al. developed the isobaric tagging (iTRAQ) method, which was quantified using MS, to improve the measurement processing capacity four-fold by injecting four samples together[3]. Then, in 2008, Sanchez et al. developed a probe called tandem mass tag (TMT) reagent, which could process six samples at once[4]. This method not only increased the multi-sample processing capacity, but also contributed to the improvement of quantitative reliability. Lipids with amino or carboxy groups are the major metabolite groups in the lipidome. As a multiplex method for lipidomics, the analysis of lipids with amino groups such as glycerophosphoethanolamine[5,6], sphingolipids[7], and gangliosides[8] has been already reported. It has been demonstrated to show very good relative quantitation by isobaric mass tagging for amino-phospholipids. Recently, Sun et al. developed TMT derivatization for fatty acids[9]. However, the target samples used were cell lines and porcine brain extracts, and the number of specimens was very limited. Therefore, we performed multiplex measurement by applying the tagging method to more than one-hundred blood samples. For this purpose, we used the LC/MS multiple reaction mode (MRM), which is widely used in targeted metabolomics. Typically, the MRM is performed on triple quadrupole (QQQ) MS and has excellent repeatability, sensitivity, and wide dynamic range, which can significantly improve quantitative accuracy[10].

Various factors related to the onset and progression of Alzheimer's disease (AD) have been investigated. Numerous reports show a relationship between lipid metabolism and AD[11]. Apolipoprotein E (APOE) plays an important role in cholesterol transport, and APOE ε4, in particular, is considered to be the most potent risk factor for AD[12],[13]. Aspirin, which suppresses the prostaglandin synthetase, cyclooxygenase, has also been reported to be effective in AD[14],[15]. Previous studies have suggested that hypercholesterolemia is a risk factor for the development of sporadic AD[16]-[17]. The precursor of amyloid peptide, amyloid precursor protein (APP), is a membrane protein, and each secretase enzyme that excises APP is also a membrane protein. There are many reports on the examination of lipids in plasma obtained from AD patients. Therefore, we prepared samples from patients with early AD and mild cognitive impairment (MCI) and from healthy elderly people to identify biomarker candidates through MRM-LC/MS after TMT derivatization.

## Materials and methods

### Reagents and human plasma samples

TMT sixplex Isobaric Label Reagent Set, Aminoxy TMT sixplex Label Reagent Set, and TMT zero Label Reagent were purchased from Thermo Fisher Scientific Inc. (Waltham, MA). Phospholipid mixtures were obtained from Avanti Polar Lipids, Inc. (Alabaster, AL) and the oleic acid standard was obtained from Sigma-Aldrich. Lipid mediators, such as eicosanoid standards, were obtained from Cayman Chemical (Ann Arbor, MI). Other reagents and solvents used were of the highest grades. Human plasma samples were purchased from two vendors, PrecisionMed Inc. (Solana Beach, CA) and Trans-Hit Bio (Quebec, Canada). PrecisionMed collected plasma from the same subjects at an average interval of one year from a total of two cohorts. Age, gender, and mini-mental state examination (MMSE) score data for patients were provided by the plasma sample vendors. Data for amyloid peptide (a-beta) 1–42 and total tau in cerebrospinal fluid (CSF) were also provided by the vendors. Pooled QC samples for each cohort were collected, mixed from each plasma sample, and divided into 15 (cohorts 1 and 2) or 10 (cohort 3) equal parts.

### Lipid extraction

Plasma samples, including a total of 40 QC samples, were extracted by methyl-tert-butyl ether (MTBE) two-phase extraction[18]. In brief, the samples (30 μL each) were added to the following organic solvents: MeOH/MTBE/H2O (225/750/188 μL) and mixed for 10 s. After a 10-min incubation at 4°C, the samples were centrifuged for phase separation. Organic upper phases were collected, and 300 μL of the solvent was dried in a vacuum centrifuge.

### TMT derivatization

**Fig 1:**
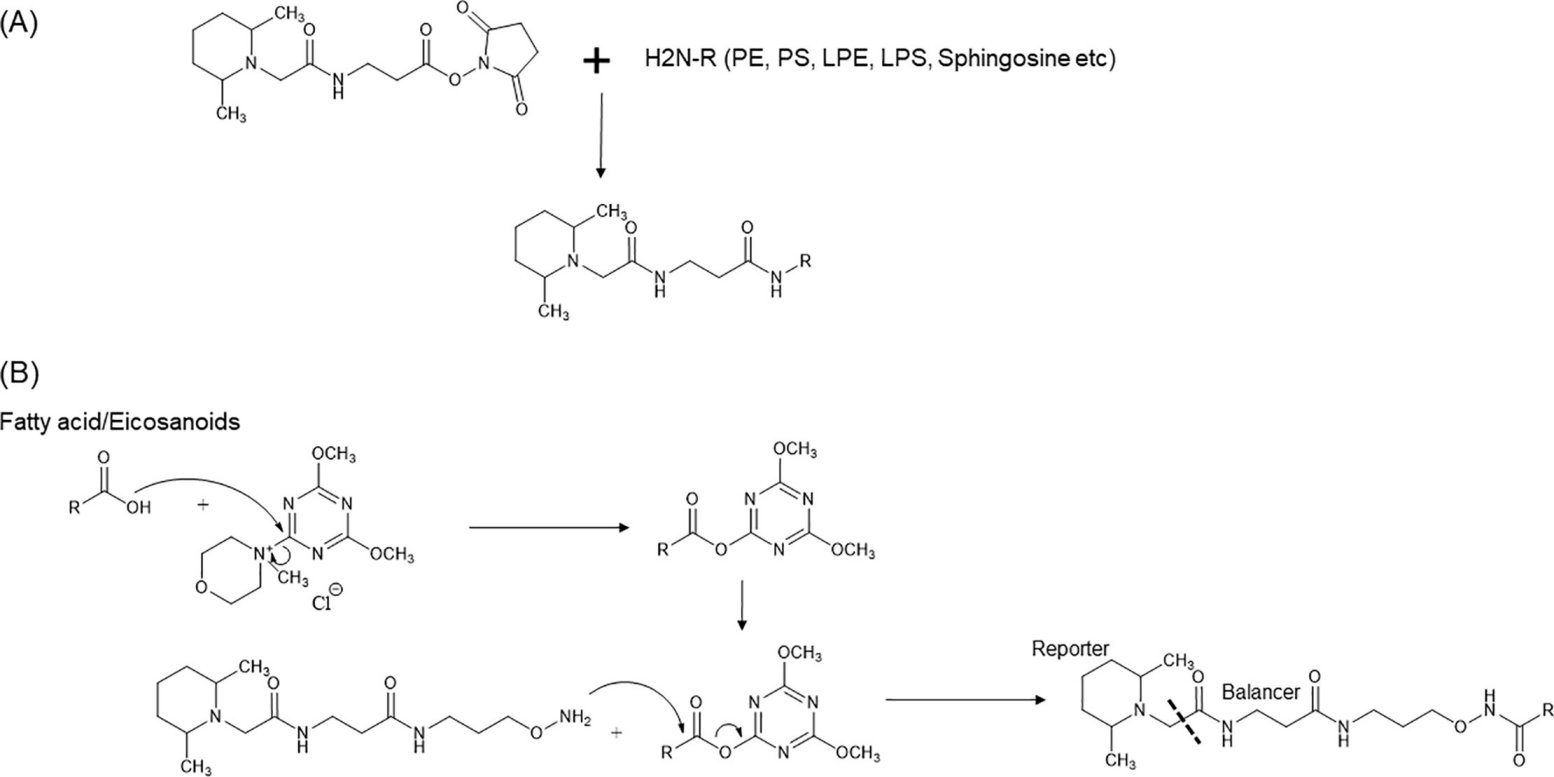
(A) Chemical reaction scheme for amino-lipids. (B) Chemical reaction scheme for carboxy-lipids.

The reaction of amino-phospholipids (phosphatidylethanolamines (PEs) or phosphatidylserines (PSs)) with the TMT reagent is shown in Fig 1A. This reagent generally reacts with primary amines under slightly alkaline conditions (pH 8–9) to form stable amide bonds; however, ester bonds of PEs and PSs are gradually hydrolyzed under alkaline conditions. However, this degradation reaction is relatively slow, and this decomposition reaction was not observed in acetonitrile or tetrahydrofuran (THF), which is an aprotic polar solvent. Thus, the MTBE-extracted sample was dissolved 50 μL of THF. Moreover, 200 μL of acetonitrile was added to the TMT reagent tube and 9 μL of the TMT solution was added to the sample tube to re-dissolve in the above THF. A reagent with 126 TMT reporter ions was added to the QC sample. The, 5 μL of triethylamine solution, diluted to 10% with THF, was added to the sample tube. After incubating at room temperature (20–25°C) for 20 h or more, 5 μL of ethanolamine solution, diluted to 10% with THF, was added to quench the reaction and incubated for 10 min or more. Then, 5 μL of acetic acid, diluted to 10% with THF, was added to neutralize the samples. The aminoxy-TMT reagent used in this study is commercially available as a derivatization reagent for the carbonyl group [19]. When linking a carboxylic acid and an amine by a stable amide bond, a condensing agent is commonly used for activating the carboxy group first, as shown in Fig 1B. Methanol (50 μL) was added to the MTBE extracted sample. NMM (4-methylmorpholine) at 11.1 μL diluted to 1% with chloroform, was added to the sample solution. DMTMM (4-(4,6-dihydroxy-1,3,5-triazin-2-yl)-4methyl morpholine) at 12 μL dissolved in methanol to produce 10 mg/ml was added to the sample tube. Acetonitrile (200 μL) was added to the aminoxy TMT reagent tube, and 9 μL of this solution was added to the sample solution. The reagent with aminoxy TMT reporter ion 126 was added to the QC sample, and other aminoxy TMT reagents (reporter ion 127–131) were added to test samples. After these three reagents were added, the solution was incubated at room temperature (20–25°C) for 4 h or more. Acetone solution (10 μL), diluted to 10% with methanol, was added to the sample solution and incubated for 10 min or more to quench the reaction. The amine groups of the amino acids were present in large amounts (total 0.1–1 mM in human plasma), compared with the activated carboxy groups, and they reacted prior to the TMT reagents. Therefore, hydrophilic substances such as amino acids were removed from human plasma as much as possible by extraction with MTBE [18], rather than simple methanol extraction. Furthermore, by activating the carboxy groups and adding the TMT reagent almost simultaneously, the excess TMT reagent preferentially reacted with the activated carboxy groups.

### LC/MS measurements

After completion of TMT derivatization, equal amounts of TMT derivatized samples were mixed to form one set of six TMT reporter ions (numbers 126–131) to prepare one sample. A total of 40 sets of 6 samples, were prepared from a total of 236 samples from 196 human plasma samples and 40 QC samples. Human plasma samples were randomized to avoid bias in any combination set. The LC and MS measurement conditions were as follows. The TMTsixplex^™^ sixplex aminoxyTMT^™^ derivatized samples were subjected to the Nexera UHPLC system and LCMS-8040 and -8060 triple quadrupole mass spectrometers (Shimadzu Co., Kyoto, Japan), respectively. The aminoxy-TMT derivatized samples were diluted twice with methanol before the measurement. An Acquity UPLC BEH C8 column (1.7 μm, 2.1 × 100 mm; Waters) was used with the following mobile phase compositions: 5 mM NH4HCO3/water (mobile phase A), acetonitrile (mobile phase B), and isopropanol (mobile phase C). The pump gradient was programmed as follows [time (%A/%B/%C)]: 0 min (95/5/0), to 8 min (70/30/0), to 16 min (30/35/35), to 28 min (6/47/47), to 35 min (6/47/47), to 35.1 min (95/5/0), and then held for 38 min for equilibration. The flow rate was 0.35 mL/min, and column temperature was 47°C. The injection volume was 5 μL. The MRM analysis was performed using the positive ion mode ESI, with a collision energy of 46 eV for aminoxy-TMT and 55 eV for TMT. For the sixplex aminoxyTMT sample, the fatty acid species and eicosanoid species possessing carbon chains with 12 to 24 carbons in length were targeted. The MRM transitions [M-H+303.25]+ →126.15, →127.15, →128.15, →129.15, →130.15, and →131.15 were used. For the sixplex TMT sample, diradyl or lyso PE and PS species with fatty acyl carbon chains 12–24 carbons in length, in sn-1 and sn-2, were targeted. SRM transitions [M+H+229.15]+ →126.15, →127.15, →128.15, →129.15, →130.15, and →131.15 were used. The peak information was obtained using Shimadzu LabSolutions (ver. 5.91). The peak areas of each individual species were normalized against the [M+H+229.15]+ →126.15 peak areas within the same phosphatidylethanolamine (PE) or phosphatidylserine (PS) species (same m/z at Q1), to determine the relative abundances. All the data were analyzed using Microsoft Excel 2016 (average, T.TEST, pearson correlation, mean square error, etc.).

## Results and discussion

Several studies have already established and reported the usefulness of isobaric mass tagging methods for lipids [5–9]. However, because this is the first application to human plasma, the reproducibility of the derivatization in plasma was confirmed by LC/MS. The MRM peak area values of each lipid were compared without correction by using pooled plasma samples. Table 1 summarizes the reproducibility of derivatization for fatty acid derivatization, and Table 2 summarizes that for PE and PS. The reproducibility results of individual lipids are shown in S1 and S2 Tables. For all lipids, CV values were within 20%, and two-thirds of lipids had CV values within 10%, indicating good reproducibility results.

**Table 1:** Reproducibility of derivatization for fatty acids in plasma (n = 6).

CV values	< 0.1	0.1–0.2	> 0.2	total
Number of fatty acids	15	6	0	21

**Table 2:** Reproducibility of derivatization for phospholipids in plasma (n = 6).

CV values	< 0.1	0.1–0.2	> 0.2	total
Number of phospholipids	28	14	0	42

**Fig 2:**
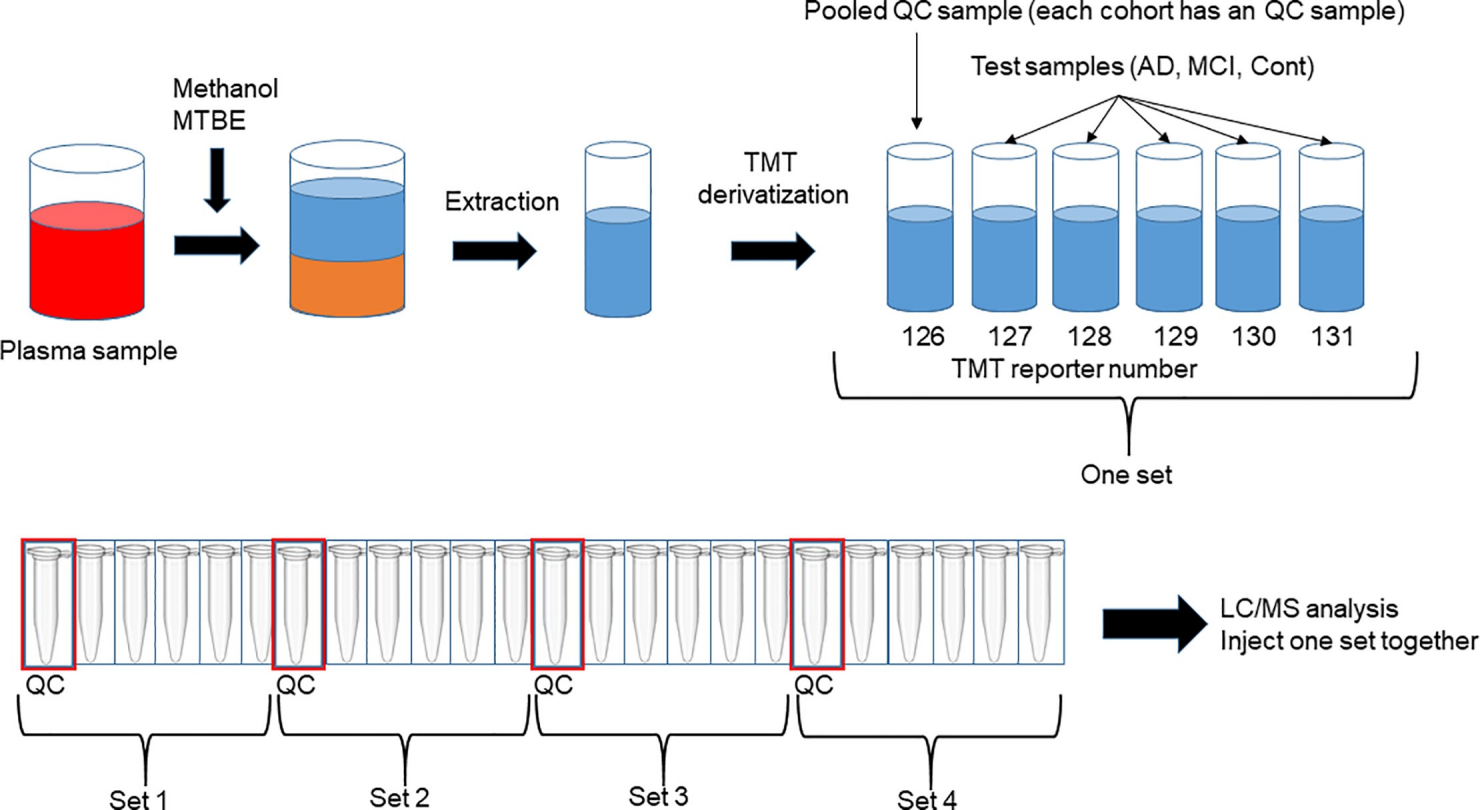
Sample process scheme.

**Fig 3:**
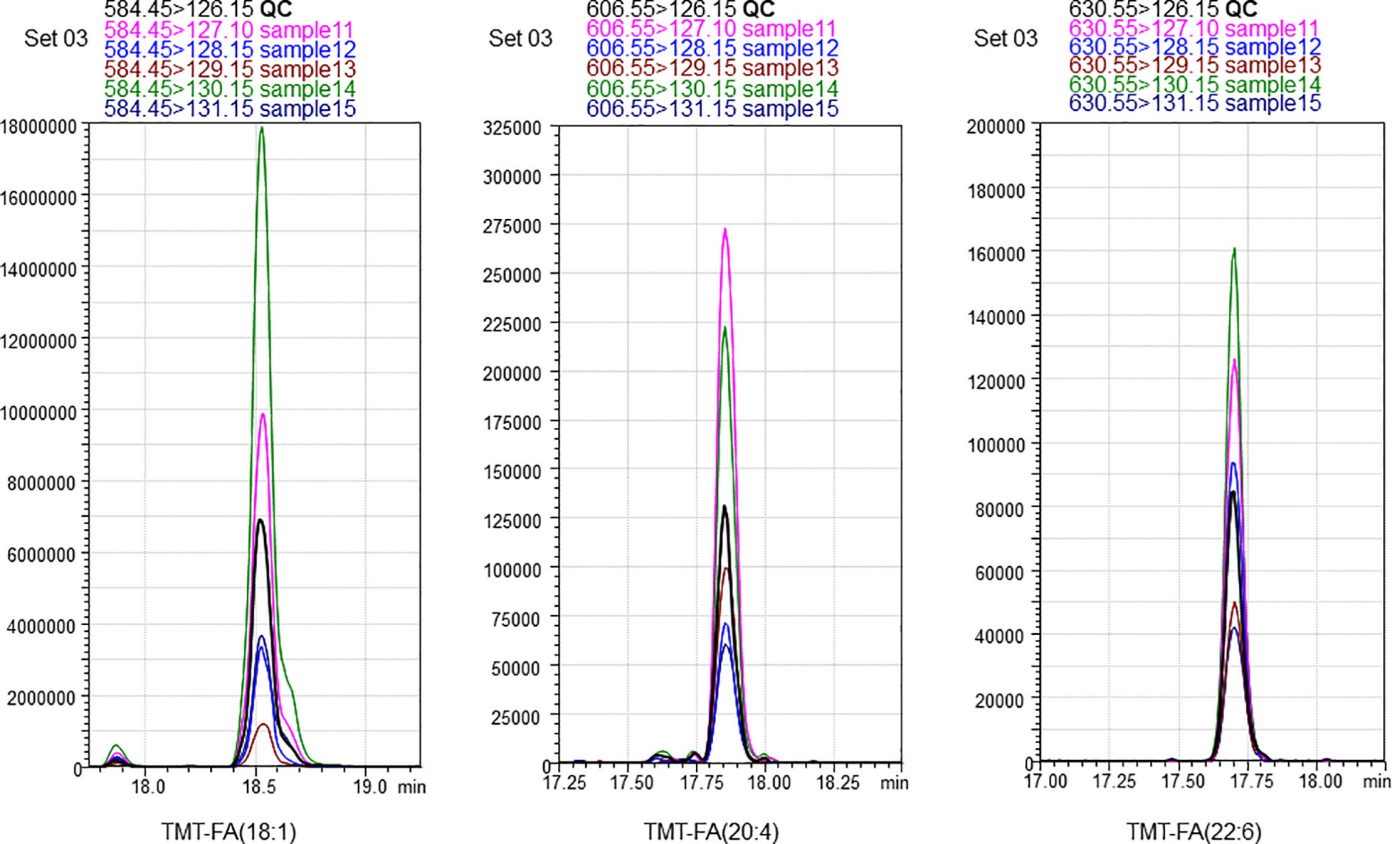
Representative MRM chromatograms of free fatty acids in cohort 1 sample set 03 after TMT derivatization. Product (TMT reporter) ions are m/z 126.15 (pooled QC sample), m/z 127.10 (MCI), m/z 128.15 (AD), m/z 129.15 (Control), m/z 130.15 (AD), and m/z 131.15 (MCI). (left) Fatty acid 18:1, precursor m/z 584.45, (middle) Fatty acid 20:4, precursor m/z 606.55, (right) Fatty acid 22:6, precursor m/z 630.55.

A total of 196 samples (Table 3) and 40 divided QC samples were collected and mixed from each sample. TMT derivatization was performed for 40 sets with 6 samples (containing 1 QC sample and 5 samples; the process scheme is shown in Fig 2). Quantification is performed using the peak intensity in MS/MS spectra in many TMT methods. The typical MRM chromatograms of TMT derivatized fatty acids in plasma are shown in Fig 3; the analytical method used for typical MRM quantification for small molecules can be used for fatty acids as well. The measurement was performed once for each set, thus improving the throughput by six-fold. Many plasma biomarker studies have been reported thus far. However, the results of many of the studies were not reproducible with different cohort samples, and thus have been made obsolete. Therefore, we verified whether the results can be reproduced even if blood samples are collected at different times, or whether the results can be similar even if blood samples are collected from different countries.

**Table 3:** Demographic characteristics of subjects in three cohorts.

	Cohort 1			Cohort 2 (follow-up of cohort 1)			Cohort 3	
	AD	MCI	Control	AD	MCI	Control	AD	Control
Number, (a-beta data*)	27 (7)	21 (11)	25 (0)	27 (13)	21 (10)	25 (0)	25 (25)	25 (25)
Female (%)	48	57	52	48	57	52	56	52
Age, mean (SD)	74.4 (10.2)	73.0 (9.2)	74.5 (3.8)	75.1 (10.2)	74.0 (9.4)	75.4 (3.9)	69.2 (7.6)	67.3 (4.4)
MMSE, mean (SD)	20.1 (3.1)	24.6 (2.2)	29.9 (0.3)	18.2 (5.7)	23.3 (3.7)	30.0 (0)	23.2 (1.5)	30.0 (0)

*Number of available a-beta 1–42 data from CSF provided by each vender

**Fig 4:**
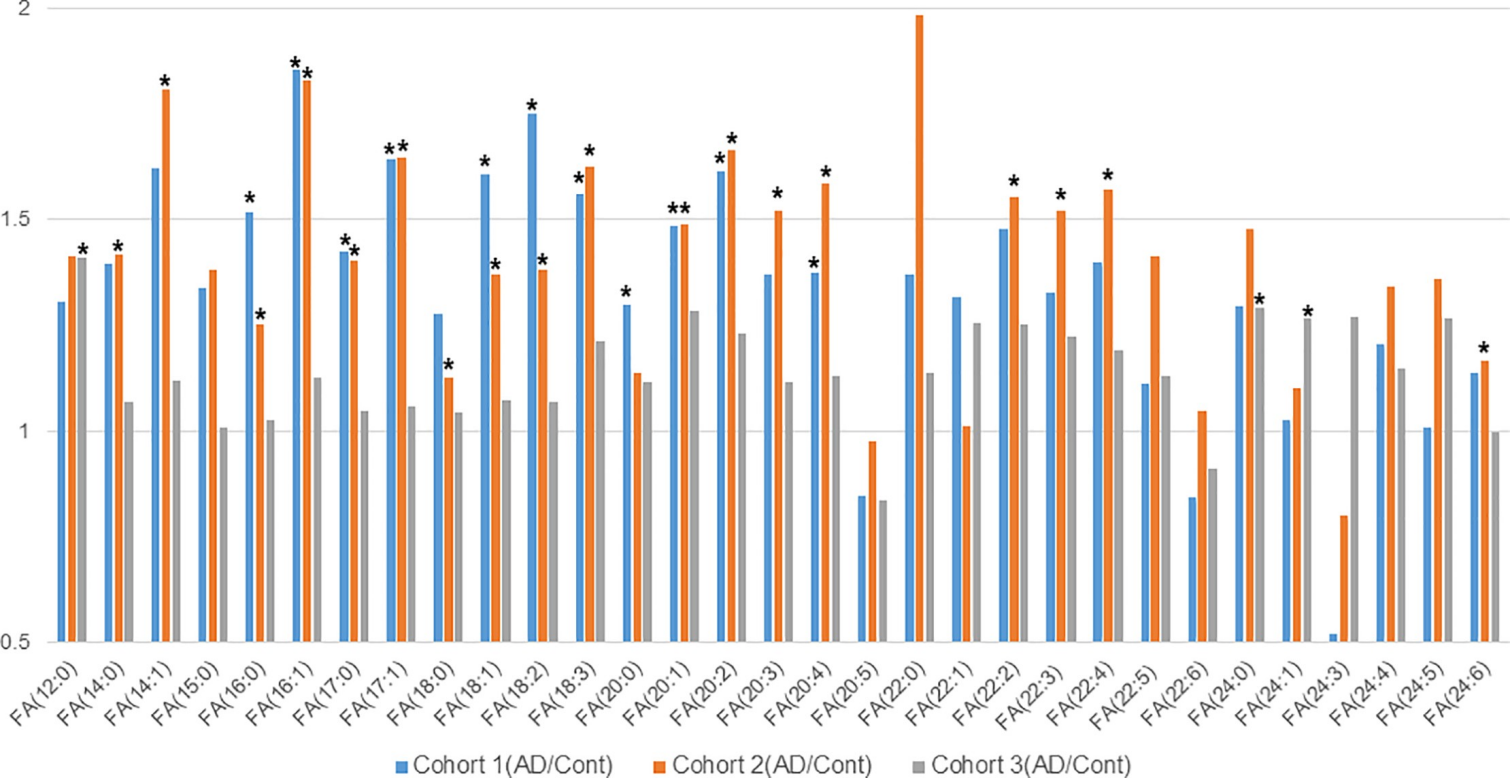
Free fatty acids levels of AD (Alzheimer’s disease) patients compared with those of Control (Cont) subjects in the three cohorts. Only statically significant *p* values (*p* < 0/05) are shown as *. Blue bars indicate AD/Cont of cohort 1, orange bars indicate AD/Cont of cohort 2, and gray bars indicate AD/Cont of cohort 3.

**Fig 5:**
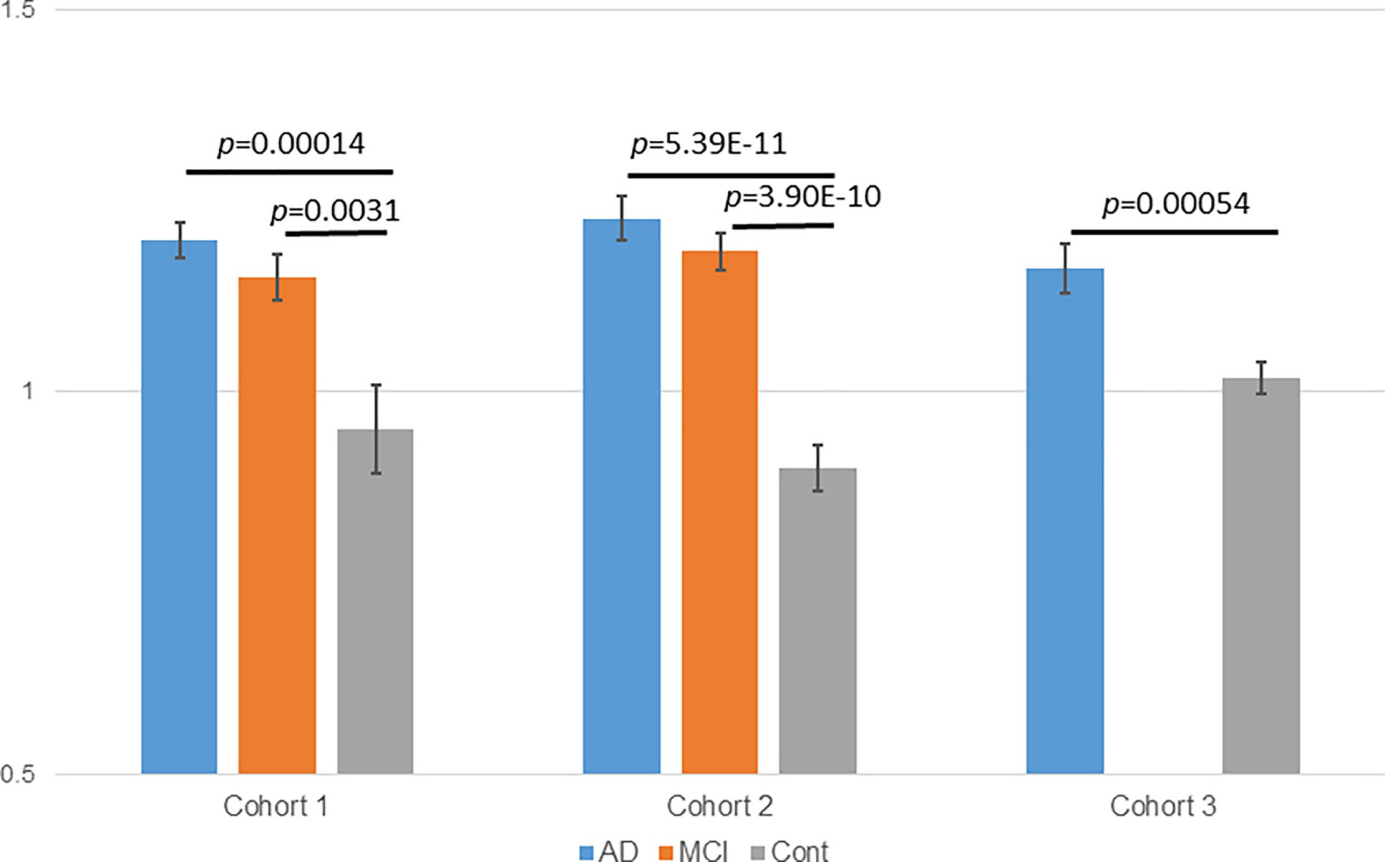
Total plasma free fatty acids in the three cohorts. Blue bars indicate AD patients, orange bars indicate MCI patients, and gray bars indicate control subjects. (left) Cohort 1, (middle) cohort 2, and (right) cohort 3. Only significant p values are shown in the graph.

The quantification was relative to the QC sample in each set; moreover, as the QC samples were common within each cohort, different datasets could be integrated. As shown in Fig 4, ten or more plasma free fatty acids significantly increased in AD and MCI in both cohort 1 and cohort 2; in cohort 3, many fatty acids showed increasing trends (the results were not significant), but only three types of free fatty acids significantly increased (individual data are given in S3–S5 Tables). This is because the samples of cohorts 1 and 2 were collected from the same subjects at different times; therefore, samples collected from the same facility from the same subjects were reproducible, but the reproducibility between facilities was not high. Nevertheless, as shown in Fig 5, compared to the total amount of free fatty acids in the plasma of the control subjects, the AD and MCI groups showed a significant increase in all cohorts. It has been reported that unsaturated fatty acids decrease in AD brains [20]; our results indicate that the amount of free fatty acids in peripheral blood increases, a result opposite to that observed in the brain. With regard to the fatty acids in plasma, a report states that DHA (FA (22: 6)) decreased in AD patients[21]; however, in that study, both covalent bound and free fatty acids were measured, whereas, here, we only measured free fatty acids, which may have caused the difference in the results.

**Fig 6:**
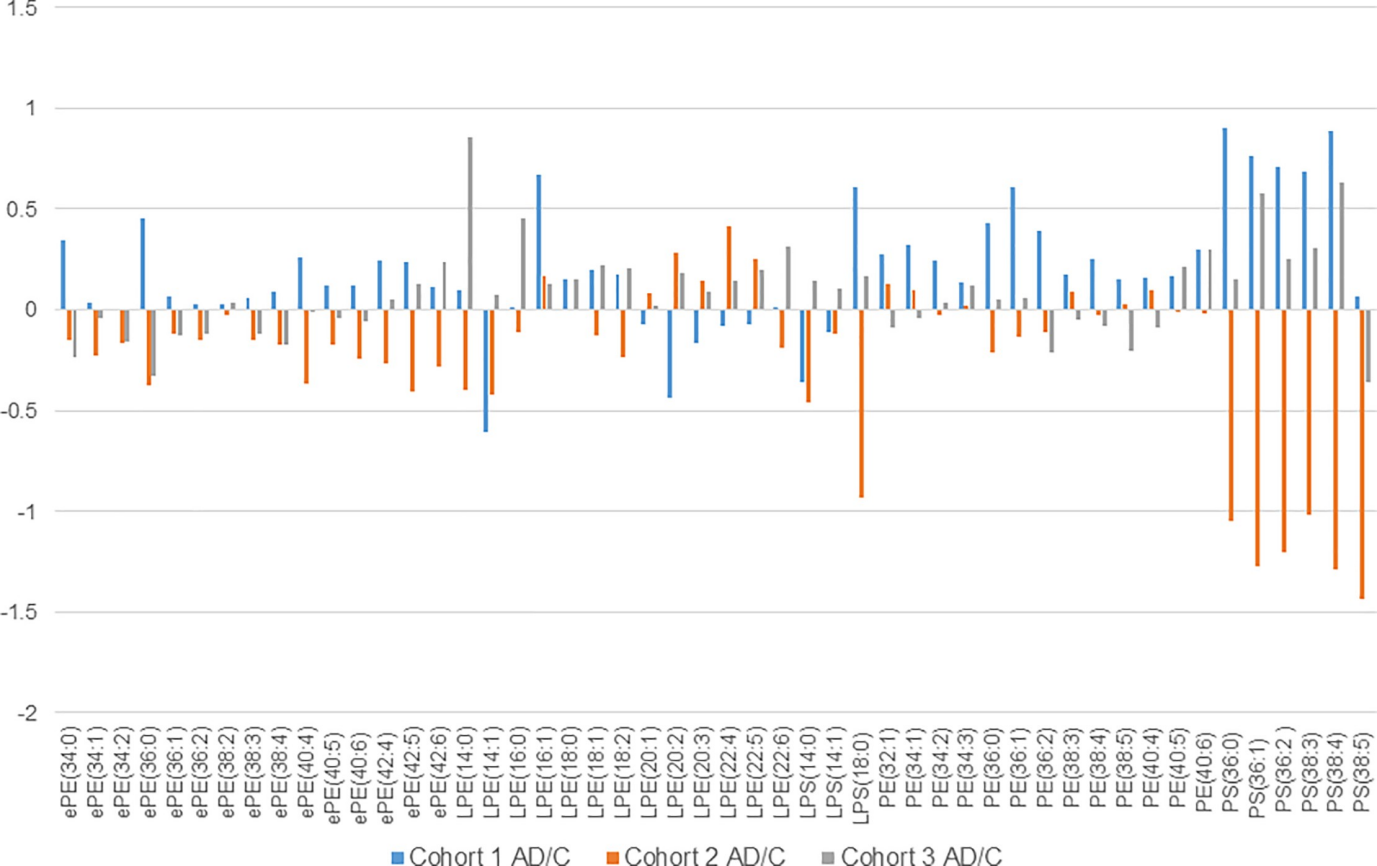
Variation in typical phospholipids between AD patients and Control (Cont) subjects among the three cohorts. Blue bars indicate AD/Cont of cohort 1, orange bars AD/Cont of cohort 2, and gray bars AD/Cont of cohort 3. The y-axis shows the logarithmic values of the AD/C ratio to the base 2.

Regarding phospholipids, several alkenyl phosphatidylethanolamines (ePEs) were significantly downregulated in AD patients compared to the control subjects, in cohort 2, and several phosphatidylserines (PSs) were statistically increased in AD patients in cohort 3. However, these trends were not reproducible among the three cohorts (Fig 6; detailed data are provided in S6–S8 Tables). These poor reproducible results are unfortunately common in the area of biomarker discovery research. However, Sato et al reported that the plasma desmosterol (precursor of cholesterol) level in AD patients was different from that in healthy subjects in both Caucasian [22] and Japanese cohorts [23]. It is important to confirm the reproducibility of data analyzing specimens obtained from different cohorts. Sato et al also found that the plasma desmosterol level varied between baseline and follow-up visits [23].

**Fig 7:**
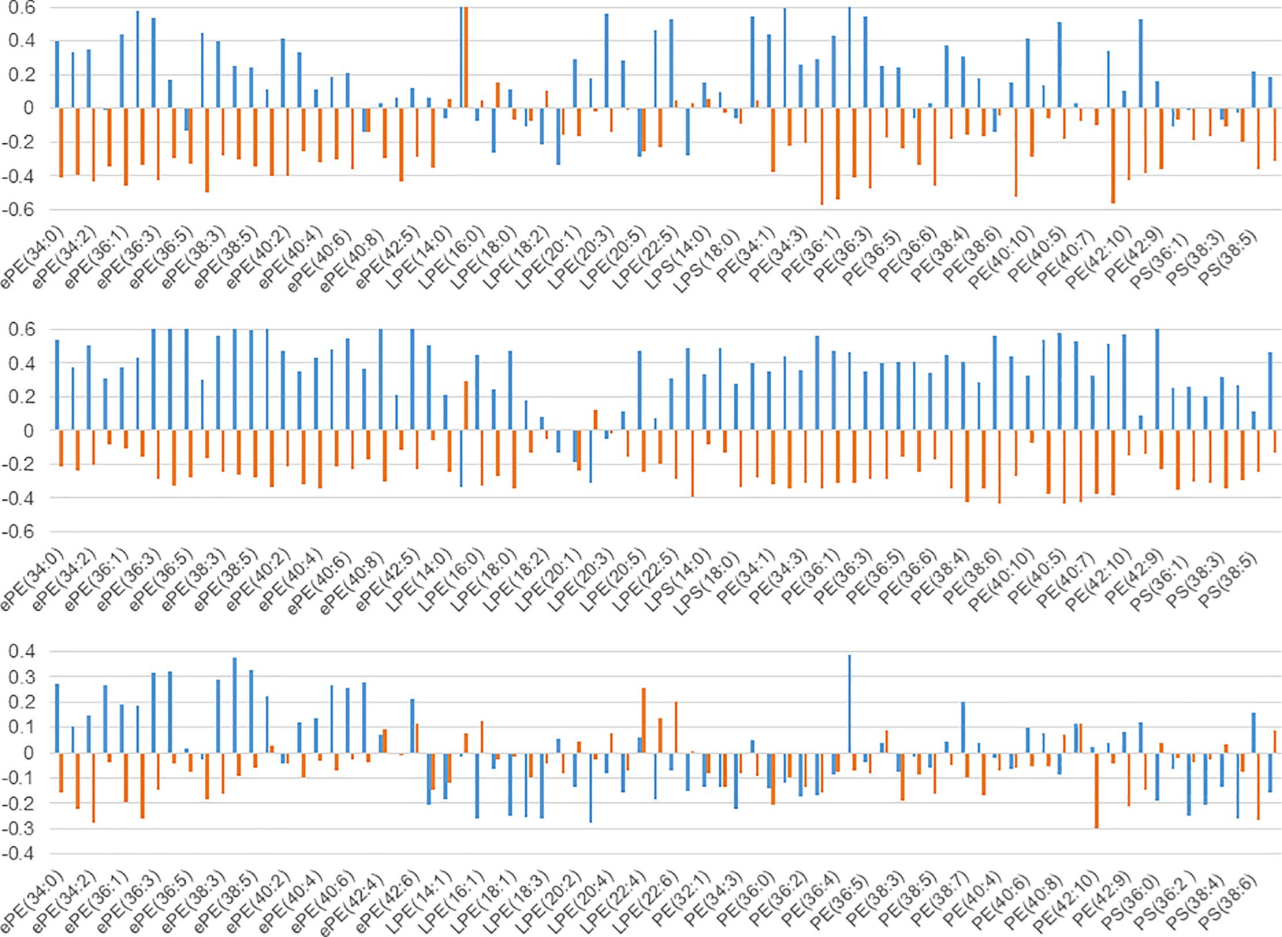
Correlation between plasma phospholipids and known CSF AD biomarkers (a-beta 1–42 or total tau). Blue bars indicate correlations between phospholipids and a-beta 1–42, and orange bars indicate correlations between phospholipids and total tau. Y-axis shows logarithm values of AD/C ratio with base 2. (upper) cohort 1, (middle) cohort 2, and (lower) cohort 3.

**Fig 8:**
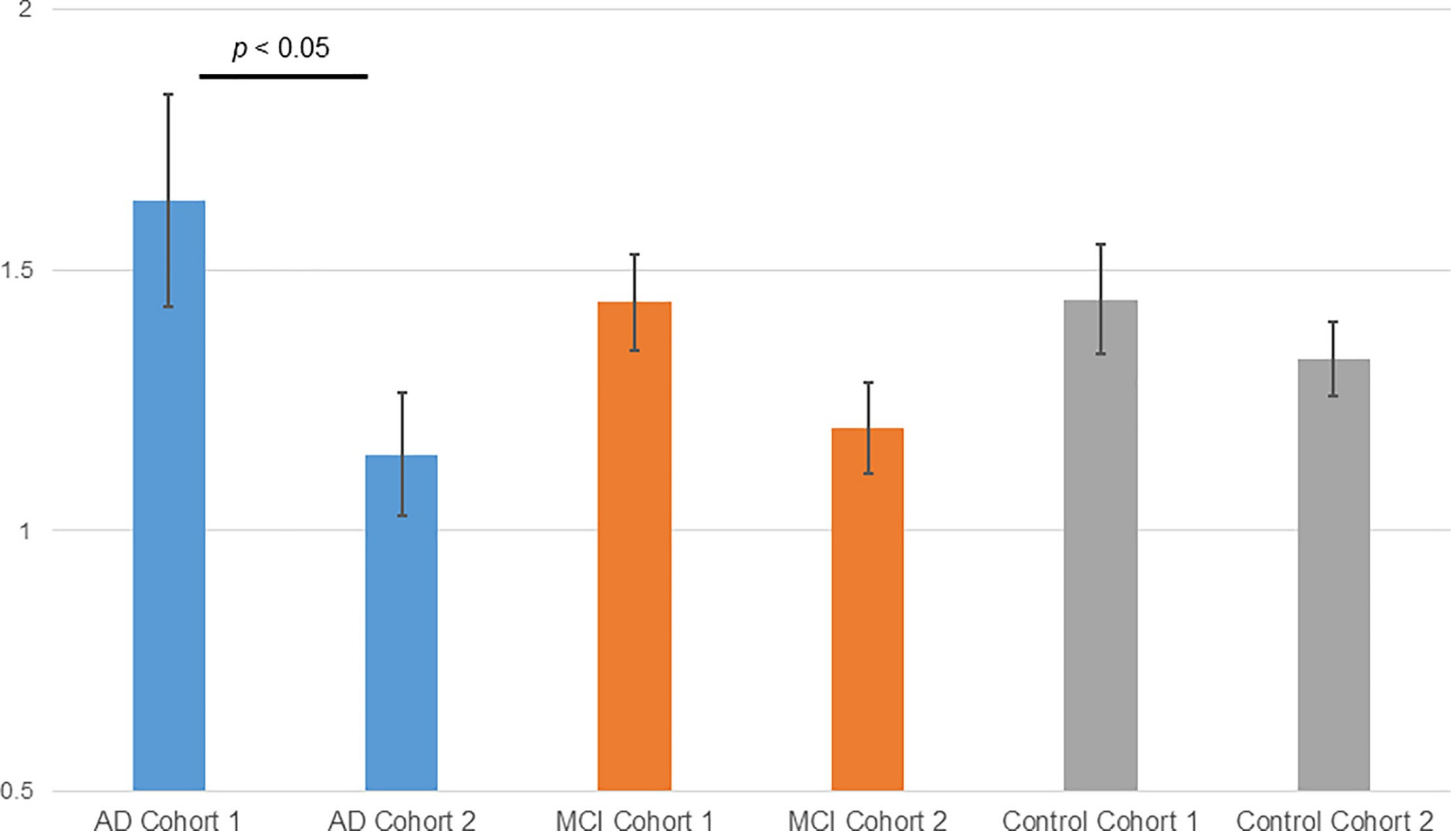
Variation in plasma ePEs between cohort 1 and cohort 2 (follow-up visit). Blue bars indicate AD patients, orange bars indicate MCI patients, and gray bars indicate control subjects.

We examined the correlation among each measured lipid molecule, age, MMSE score, a-beta 1–42, and total tau in the CSF in each cohort. Many ePEs had a very weak positive correlation (>0.2) with CSF a-beta 1–42 and a very weak reversed correlation (< - 0.2) with total tau in the CSF in all three cohorts (Fig 7); however, other phospholipids did not show reproducible results among the three cohorts. Although the cause of AD is still unknown, amyloid peptide and tau protein are likely to be related to AD. Therefore, ePEs may be related to AD, because the biomarkers a-beta 1–42 and total tau were weakly correlated with ePEs in the blood samples from the three cohorts. Therefore, we measured variations in the ePE levels in the same subject after approximately one year and compared the values between cohorts 1 and 2. Fig 8 shows a comparison of the total ePE levels in patients with AD in cohort 1 with those in cohort 2. The total ePE levels decreased in cohort 2 during the one-year follow-up visit; however, no significant variations were observed in the control group. ePEs, thus, correlated with CSF a-beta 1–42, and the expression levels decreased only in patients with AD (time course). The ePEs vary considerably across lipid molecules, and the difference in their levels between disease and control samples was not clear for any single time point (Fig 6); however, it may be beneficial to determine the difference in total ePEs, not individual ePEs, between patients with AD and healthy controls, if follow-up samples are available. To date, ePE (plasmalogen) has been shown to decrease in AD [24], and this result is consistent with our data. Furthermore, the serum plasmalogen level decreases in patients with AD as the degree of reduced recognition increases [25], which is also consistent with our result. Unfortunately, samples from the same subjects at different times were only collected for cohorts 1 and 2, and the reproducibility of the results showing the changes could not be verified. Nevertheless, since ePE levels vary in early AD, it can be used as a monitoring marker before and after the onset of AD. In contrast, the levels of plasma fatty acids do not vary during a one-year interval, and free fatty acid levels did not correlate with MMSE scores. However, the total free fatty acid levels were high in patients with AD in all three cohorts. Therefore, plasma free fatty acid levels may be a risk marker for AD development.

Mapstone et al. found that ten plasma lipids could predict conversion to MCI or AD, and eight lipids out of ten were phosphatidylcholines (PCs)[26]. PCs are the most abundant lipids, and would provide reliable and reproducible data because of the strong peaks generated on LC/MS. In plasma proteomics, eliminating abundant proteins like albumin is crucial for identifying promising plasma biomarkers. Casanova et al. failed to replicate the results of Mapstone’s data by analyzing different cohort samples [27]; however, this was an expected outcome due to a difference in cohort samples. There are several factors in human plasma that may influence biomarker data. In this study, we focused on PE, PS, and fatty acids, which may be useful for mitigating the errors caused by abundant lipids, i.e., PCs, while TMT labeling provides more data owing to increased throughput from multiple cohort analysis. The three cohorts confirmed that the amount of free fatty acids in plasma was already increased in the early AD or MCI stage and that ePEs were correlated with CSF a-beta 1–42. Furthermore, plasma ePEs decreased significantly in the one-year interval in AD patients, however, did not vary in healthy subjects. In this study, each group was as small as 21–27 specimens; cohort 2 was a follow-up of cohort 1, and cohort 3 was obtained from a country different from those of cohorts 1 and 2; therefore, it was important to confirm that the three cohorts could show reproducible results. In addition, the presence of relatively early AD patients at this time indicated that these plasma lipids were good biomarkers for early diagnosis of AD. However, given the improved throughput, we believe that it would be relatively easy to scale up to several orders of samples to obtain more concrete results. Moreover, it is not necessary to prepare a large number of internal standard substances for this method, correction to QC samples is easy, and rapid quantitative comparison using previously reported isobaric mass tagging method can be achieved [5][6][7][8][9]. It would be ideal to obtain a large-scale pool of QC plasma samples for global QC across all cohort samples. Furthermore, MRM measurement by QQQ MS is a suitable quantification method. There are many studies on the quantitative analysis, as drug analysis, of low molecular weight compounds such; thus, the combination of TMT and MRM is a powerful technique for lipid analysis. Several TMT users conduct information-dependent acquisition (IDA) (or DDA–data-dependent acquisition) using an Orbitrap MS or a Q-tof MS. The drawback in this case is that if there are multiple measurements, it is not always possible to acquire an MS scan for the same precursor ion each time [28]. Therefore, if there are 40 measurements, as in this study, there is a risk that a similar dataset will not be obtained for all measurements, with some missing data. However, MRM measurements with QQQ MS, which is pre-targeted, can result in obtaining the same dataset for all measurements. For TMT analysis with Orbitrap MS or Q-tof MS, the advantage is that the peak intensity from MS does not saturate even if the precursor ion intensity is extremely strong, while the MRM approach is sensitive to signal saturation for targets with high expression levels. Current bottlenecks in biomarker discovery are obtaining a large number of plasma specimens with detailed clinical information. However, we believe that TMT-lipidomics allows the analysis of large-scale cohort samples.

## Supporting information

S1 Table Reproducibility of TMT reaction efficiency for fatty acids (FAs) in plasma. (XLSX)

S2 Table Reproducibility of TMT reaction efficiency for phospholipids in plasma. (XLSX)

S3 Table Individual free fatty acids and subject data in cohort 1. (XLSX)

S4 Table Individual free fatty acids and subject data in cohort 2. (XLSX)

S5 Table Individual free fatty acids and subject data in cohort 3. (XLSX)

S6 Table Individual phospholipids and subject data in cohort 1. (XLSX)

S7 Table Individual phospholipids and subject data in cohort 2. (XLSX)

S8 Table Individual phospholipids and subject data in cohort 3. (XLSX)

S9 Table Individual fatty acids data before normalization including MRM settings, retention time and peak area in cohort 1. (XLSX)

S10 Table Individual fatty acids data before normalization including MRM settings, retention time and peak area in cohort 2. (XLSX)

S11 Table Individual fatty acids data before normalization including MRM settings, retention time and peak area in cohort 3. (XLSX)

S12 Table Individual phospholipids data before normalization including MRM settings, retention time and peak area in cohort 1. (XLSX)

S13 Table Individual phospholipids data before normalization including MRM settings, retention time and peak area in cohort 2. (XLSX)

S14 Table Individual phospholipids data before normalization including MRM settings, retention time and peak area in cohort 3. (XLSX)
